# Categorization of a Universal Coding System to Distinguish Use of Durable Medical Equipment and Supplies in Pediatric Patients

**DOI:** 10.1001/jamanetworkopen.2023.39449

**Published:** 2023-10-24

**Authors:** Arda Hotz, Eli Sprecher, Lucia Bastianelli, Jonathan Rodean, Isabel Stringfellow, Elizabeth Barkoudah, Laurie E. Cohen, Carlos Estrada, Robert Graham, Jonathan Greenwood, Jennifer Kyle, Nina Mann, Maria Pinkham, Toni Solari, Rachel Rosen, Susan Saleeb, Ankoor S. Shah, Karen Watters, Sarah Wells, Jay G. Berry

**Affiliations:** 1 Complex Care, Division of General Pediatrics, Boston Children’s Hospital, Boston, Massachusetts; 2 Department of Pediatrics, Harvard Medical School, Boston, Massachusetts; 3 Cerebral Palsy and Spasticity Center, Boston Children's Hospital, Boston, Massachusetts; 4 Children’s Hospital Association, Lenexa, Kansas; 5 Department of Neurology, Boston Children’s Hospital, Boston, Massachusetts; 6 Department of Neurology, Harvard Medical School, Boston, Massachusetts; 7 Division of Pediatric Endocrinology & Diabetes, The Children’s Hospital at Montefiore, Bronx, New York; 8 Department of Pediatrics, Albert Einstein College of Medicine, Bronx, New York; 9 Department of Urology, Boston Children’s Hospital, Boston, Massachusetts; 10 Department of Surgery, Harvard Medical School, Boston, Massachusetts; 11 Critical Care Medicine, Department of Anesthesiology, Critical Care, and Pain Medicine, Boston Children’s Hospital, Boston, Massachusetts; 12 Department of Anaesthesia, Harvard Medical School, Boston, Massachusetts; 13 Department of Physical Therapy and Occupational Therapy Services, Boston Children’s Hospital, Boston, Massachusetts; 14 UnitedHealthcare, Minneapolis, Minnesota; 15 Division of Nephrology, Boston Children’s Hospital, Boston, Massachusetts; 16 Division of Gastroenterology, Hepatology, and Nutrition, Boston Children’s Hospital, Boston, Massachusetts; 17 Department of Cardiology, Boston Children’s Hospital, Boston, Massachusetts; 18 Department of Ophthalmology, Boston Children’s Hospital, Boston, Massachusetts; 19 Department of Ophthalmology, Harvard Medical School, Boston, Massachusetts; 20 Department of Otolaryngology, Boston Children’s Hospital, Boston, Massachusetts; 21 Department of Otolaryngology, Harvard Medical School, Boston, Massachusetts

## Abstract

**Importance:**

Although durable medical equipment and supplies (DMES) are commonly used to optimize the health and function in pediatric patients, little is known about the prevalence of use and spending on DMES.

**Objective:**

To categorize the Healthcare Common Procedure Coding System (HCPCS) for distinguishing DMES types, and to measure the prevalence and related spending of DMES in pediatric patients using Medicaid.

**Design, Setting, and Participants:**

This study is a cross-sectional analysis of the 2018 Merative Medicaid Database and included 4 569 473 pediatric patients aged 0 to 21 years enrolled in Medicaid in 12 US states from January 1 to December 31, 2018. Data were analyzed from February 2019 to April 2023.

**Exposure:**

DMES exposure was identified with the Centers for Medicare & Medicaid Services HCPCS codes. Three pediatricians categorized HCPCS DMES codes submitted by vendors for reimbursement of dispensed DMES into DMES types and end-organ systems; 15 expert reviewers refined the categorization (2576 DMES codes, 164 DMES types, 14 organ systems).

**Main Outcomes and Measures:**

The main outcome was DMES prevalence & Medicaid spending. The χ^2^ test was used to compare DMES prevalence and Wilcoxon rank sum tests were used to compare per-member-per-year (PMPY) spending by complex chronic conditions (CCC).

**Results:**

Of the 4 569 473 patients in the study cohort, 49.3% were female and 56.1% were aged 5 to 15 years. Patients used 133 of 164 (81.1%) DMES types. The DMES prevalence was 17.1% (95% CI, 17.0%-17.2%) ranging from 10.1% (95% CI, 10.0%-10.2%) in patients with no chronic condition to 60.9% (95% CI, 60.8%-61.0%) for patients with 2 or more CCCs. The PMPY DMES spending was $593, ranging from $349 for no chronic condition to $4253 for 2 or more CCCs. Lens (7.9%), vision frames (6.2%), and orthotics for orthopedic injury (0.8%) were the most common DME in patients with no chronic condition. Enteral tube / feeding supplies (19.8%), diapers (19.2%), lower extremity orthotics (12.3%), wheelchair (9.6%), oxygen (9.0%), and urinary catheter equipment (4.2%) were among the most common DMES in children with 2 or more CCCs.

**Conclusions and Relevance:**

In this cross-sectional study, HCPCS distinguished a variety of DME types and use across pediatric populations. Further investigation should assess the utility of the HCPCS DMES categorization with efforts to optimize the quality and safety of DMES use.

## Key Points

**Question:**

How well does the Healthcare Common Procedure Coding System (HCPCS) distinguish use of durable medical equipment and supplies (DMES) in pediatric patients?

**Findings:**

In this cross-sectional study including 4 569 473 pediatric patients enrolled in Medicaid, 133 of 164 (81%) DMES types were used in the study sample. The highest use of DMES (60%) occurred in children with multiple complex chronic conditions.

**Meaning:**

Results of this study suggest that HCPCS distinguished a variety of DMES; further investigation in the quality and safety of DMES administration and use is warranted.

## Teaser

This cross-sectional study identifies and categorizes durable medical equipment and supplies within the Healthcare Common Procedures Coding System and compares the prevalence, type, and spending used in different populations of children.

## Introduction

Pediatric patients rely on durable medical equipment and supplies (DMES) to overcome limitations in physiologic function, thereby promoting maximal development. For example, they use orthopedic braces to overcome acute musculoskeletal injury, corrective lenses to mitigate vision impairment and prevent vision maldevelopment, and noninvasive positive pressure ventilation (NIPPV) to alleviate obstructive sleep apnea. Some children and youth with special health care needs (CYSHCN) rely on so many DMES that *hospital in the home* best describes their residential environment.^1,2^ An emerging body of literature suggests that the population of children who rely on specific DMES, including for gastrostomy and tracheostomy, may be increasing.^3,4^

Little is known about the epidemiology and health care spending of DMES use in children, including rates of use across healthy children and CYSHCN. In studies of elderly Medicare beneficiaries from years 2004-2006, 4% used DMES and DMES spending averaged $470 annually per patient.^5^ Challenges with identifying DMES use in populations of children has been a major research limitation. US state-based research projects from prior decades measured use of a limited set of DMES through surveys to families.^6,7^ Payor data on administrative claims of DMES may be a useful source of information. Claims for DMES that are dispensed to patients for use in their home environment are itemized and processed using the Centers for Medicare & Medicaid Services (CMS) open-source Level II Healthcare Common Procedure Coding System (HCPCS).^8^ The HCPCS contains clinically rich information on DMES, spread across 17 Level II sections.^9^ Most DMES codes contain sufficient information to identify the type of DMES used (eg, mechanical ventilator) and to distinguish the organ system that correlates with the type of DMES used (eg, respiratory system for mechanical ventilator).

The HCPCS has been used in prior studies^3,10,11^ to assess specific types of DMES use and spending related to respiratory and cancer care in children. These studies have reported that HCPCS substantially augments traditional *International Classification of Diseases* diagnosis and procedure billing codes by identifying DMES use. However, the current HCPCS does not contain hierarchical groupings to distinguish all types of DMES or the body organ system that the DMES is intended to help. Such groupings would permit measurement of DMES use and spending across all types and organ systems. Therefore, we performed the current study to (1) identify and categorize DMES within the HCPCS Level II Codes and (2) compare the prevalence, types, and spending of DMES used by different populations of children, including healthy children and CYSHCN.

## Methods

This retrospective cross-sectional study of deidentified administrative claims data was exempt from Boston Children’s Hospital institutional review board as not human participant research. The study follows the Strengthening the Reporting of Observational Studies in Epidemiology (STROBE) reporting guideline for observational studies. This cross-sectional analysis of the 2018 Merative Medicaid Database included 4 569 473 pediatric patients aged 0 to 21 years enrolled in Medicaid in 12 US states from January 1 to December 31, 2018. Data were analyzed from February 2019 to April 2023.

### Initial HCPCS Categorization

To begin HCPCS categorization, 2 general pediatricians (AH and ES) with extensive clinical experience managing a broad range of DMES in children independently reviewed ~ 10 000 HCPCS Level II alphanumeric codes along with their corresponding short and long descriptions to distinguish and categorize DMES. Discrepancies were resolved with a third pediatrician reviewer (JB). DMES were defined as replaceable medical equipment or supplies (disposable or nondisposable) issued to a patient in their home to address a medical need or function. HCPCS codes for medications, surgery/procedure equipment, and clinician health services (eg, physical therapy provided in a patient’s home) were not considered DMES. For each HCPCS code considered DMES, both a specific DMES type and an organ system was assigned. (Table 1) Each code was assigned only 1 specific DMES and to 1 organ system. For example, 6 HCPCS codes were assigned to specific DMES *mechanical ventilator* under the encompassing organ system *respiratory*.

**Table 1.** Prevalence and Types of DMES in Pediatric Patients Using Medicaid

DMES category	Prevalence per 1000 enrollees	Most common DMES types
Ophthalmologic	114.2	Frame lens, vision frames
Musculoskeletal	29.4	Orthopedic injury (ie, crutches), orthotic lower extremity
Respiratory	26.9	Nebulizer, aerochamber/peak flow
Other	6.6	Breast pump, gloves
Genitourinary	5.9	Diapers, underpads
Nutrition	5.1	Formula, nutrition (additive)
Skin and soft tissue	3.7	Skin dressing, disinfectant
Gastroenterological	2.2	Enteral supplies, enteral tube
Ear, nose, and throat	1.6	Assisted hearing, cochlear implant
Endocrine	1.1	Continuous glucose monitoring, insulin pump
Vascular	0.6	Infusion supplies, indwelling port
Neurologic	0.1	Neurostimulator (external), neurostimulator (implantable)
Renal	0.1	Blood pressure, dialysis
Cardiac	<0.1	Pacemaker, defibrillator (external)

Abbreviation: DMES, durable medical equipment and supplies.

### Refinement of HCPCS Categorization

Thirteen pediatric specialty clinicians (E.B., L.C., C.E., R.G., J.G., N.M., M.P., T.S., R.R., S.S., A.S., K.W., and S.W.), 1 insurance representative (J.K.), and 1 patient/family representative (L.B.) reviewed and refined the DMES codes into specific types within organ systems to optimize accuracy and nomenclature. We selected these experts based on their extensive experience with DMES use in children, ensuring that—collectively—the experts covered all of the clinical areas represented in the initial HCPCS categorization of DMES type and organ system. The clinicians practiced in the following fields: cardiology, critical care pulmonology, endocrinology, gastroenterology, nephrology, neurology, nutrition, ophthalmology, otolaryngology, physical therapy, pulmonology, urology, and wound care. Proposed revisions were appraised and affirmed by AH, ES, and JB, through a two-thirds majority vote. eTable 1 and eTable 2 in Supplement 1 and the eAppendix in Supplement 2 present technical details on DMES categorization methods.

### Testing the Categorized HCPCS for DMES Use in Children

The refined categorized HCPCS was applied to the 2018 Merative Medicaid Multistate Database. This database includes health care claims across the continuum (eg, DMES vendors, inpatient, outpatient) for Medicaid enrollees in 12 US states. Inclusion criteria for the study population was age 0 to 21 years and continuous enrollment in Medicaid for at least 11 of 12 months. The data usage agreement blinded the US states for identification.

### Main Outcome Measure

Presence of a DMES claim was the main outcome, identified from HCPCS categorization system. The secondary outcome was standardized Medicaid spending for DMES. For each DMES category and type, spending was calculated in aggregate and by per-member-per-year (PMPY). Because Medicaid spending may not be itemized for enrollees in managed care plans, we imputed itemized spending measured from fee-for-service encounters for the same health service.

### Patient Clinical and Demographic Characteristics

Because a heterogeneous pediatric population use DMES, participant status was classified as with or without a chronic condition using *International Statistical Classification of Diseases, Tenth Revision, Clinical Modification (ICD-10-CM)* diagnosis codes from the Agency for Healthcare Research and Quality Chronic Condition Indicator System.^12^ We further classified chronic condition in pediatric patients as a complex chronic condition using *ICD-10-CM* diagnosis codes from the Complex Chronic Condition system (v2) of Feudtner et al.^13^

### Statistical Analysis

We compared prevalence as well as type and number of DMES used by the pediatric patients’ chronic condition classification (no chronic condition, noncomplex chronic condition [1 and 2 or more], complex chronic condition [1 and 2 or more]) and their demographic characteristics using χ^2^ tests. We calculated total aggregate spending on DMES across the study population as well as PMPY Medicaid spending (ie, total spending on DMES divided by the number of children using DMES) on DMES across chronic condition groupings and compared per-year spending distributions across groups with Wilcoxon rank sum test. For statistical significance, we used a 2-sided hypothesis test with α = .05. Data were analyzed using SAS version 9.4 (SAS Institute Inc).

## Results

### Categorization of HCPCS

The initial categorization produced 161 DMES types with 15 organ systems, which were refined to 164 types and 14 organ systems after expert review (eTable 1 in Supplement 1). Also, during refinement, 151 of 2576 total DMES codes (6%) were changed to a different end-organ system category (eg, HCPCS E0606 *postural drainage board* was changed by system classification from *other* to *respiratory* system), suggesting 94% agreement with the initial DME system categorization; and 205 DMES codes (8%) were changed to a different or new DMES type within an end-organ system category (eg, HCPCS E0606 changed from *drainage board* to *airway clearance*), suggesting 92% agreement with DMES type categorization.

### Prevalence of DMES Use in Pediatric Patients Using Medicaid

Of the 4 569 473 pediatric patients in the Medicaid analytical cohort, 49.3% were female and 56.1% were age 5 to 15 years. Seventeen percent (n = 782 425) had at least 1 DME claim. Eleven percent used ophthalmologic DME only; 6 percent of children used another type of DME, including musculoskeletal (2.9%) and respiratory (2.7%) (Table 1). Nebulizer for respiratory treatments (1.9%) and orthotics for mobility (eg, braces) (1.2%) were the most common specific types of DME used in the respiratory and musculoskeletal systems, respectively. Cardiac, neurologic, renal, and vascular were among the least common DMES categories, used in less than 1 per 10 000 children. (Table 1)

### Spending on DMES Use in Pediatric Patients Using Medicaid

**Figure 1.**
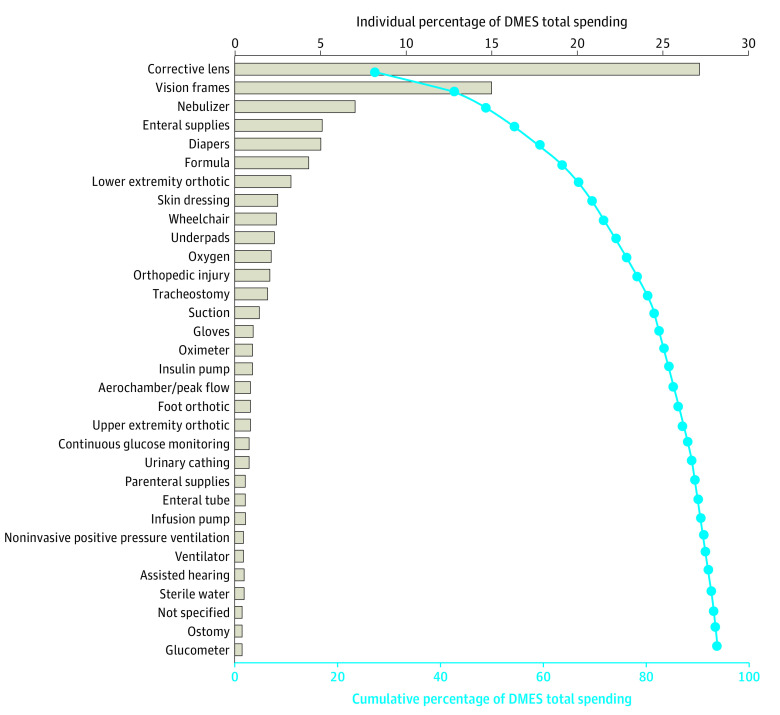
Allocation of Medicaid Spending on Durable Medical Equipment and Supplies (DMES) in Pediatric Patients The bars indicate the percentage of total DMES spending for each DMES. The connected dots indicate the cumulative percentage of total Medicaid spending accumulated across multiple DMES. Corrective lens includes contact, frame, and scleral lens. Cath indicates catheterization.

DMES aggregate spending across the study states was $464.2 million (US), which accounted for 3.4% of total Medicaid spending on all health services for all pediatric patients in the study cohort. The PMPY DMES spending for DMES users was $593 ($464.2 million divided by 782 425 patients using DMES). The PMPY DMES spending across the entire study population (DME users and nonusers) was $102. The largest proportions of aggregate DME spending across all patients included lens ($126.0 million; 27.1%), vision frames ($69.6 million; $15.0%), nebulizer ($32.4 million; 7.0%), enteral supplies ($23.8 million; 5.1%), and diapers ($23.2% million, 5.0%). (Figure 1) These 5 DMES types accounted for 59.3% of total Medicaid spending on DMES. DMES with the highest PMPY included parenteral supplies (n = 192 children [<0.1%]; PMPY = $14 745), tracheostomy (n = 2686 children [0.1%]; PMPY = $3200), infusion pump (n = 1491 children [<0.1%]; PMPY = $1764), skin wound care (n = 249 children [<0.1%]; PMPY = $1663), and oxygen (n = 5874 children [0.1%]; PMPY = $1646).

### DMES Use and Spending in Patients With and Without Chronic Conditions

**Figure 2.**
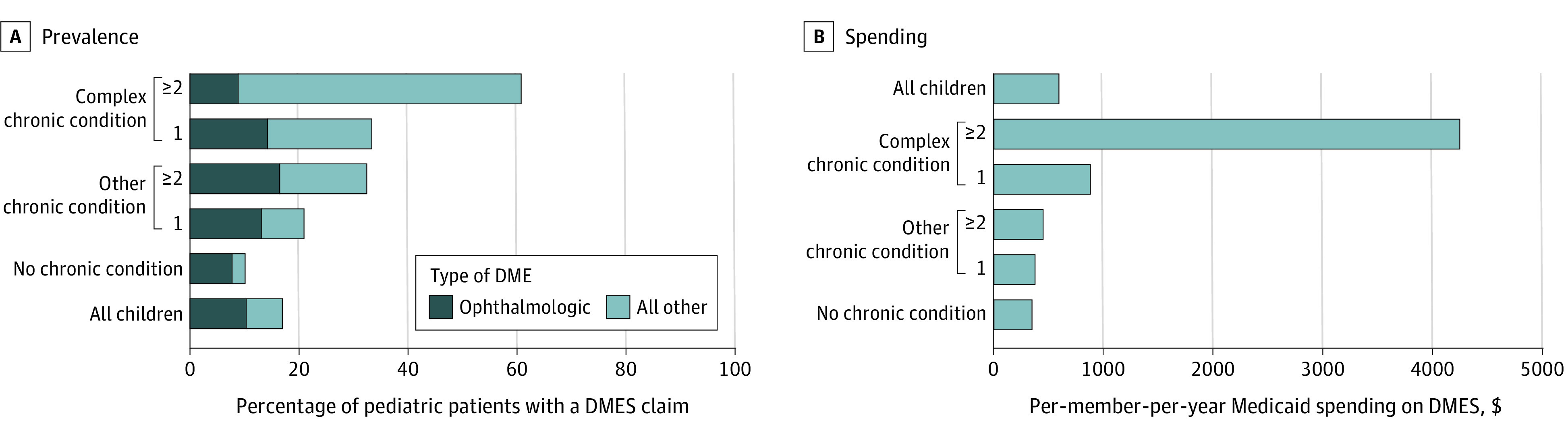
Prevalence and Spending on Durable Medical Equipment and Supplies (DMES) by Type and Number of Chronic Conditions in Pediatric Patients Using Medicaid A, The percentage of children with a DMES health care claim varied significantly with χ^2^ test by chronic condition category. B, The per-member-per-year spending was calculated by dividing the total aggregate spending on DMES for each patient group by the number of children who used DMES in each group.

Ten percent of pediatric patients without a chronic condition used DMES (Figure 2) and accounted for 20.0% ($92.9 million) of all DMES spending. Among these patients without chronic conditions who used DMES, 80% of this subgroup had ophthalmologic DMES (lenses and frames). Twenty-six percent of children with a chronic condition used DMES and accounted for 80.0% ($371.3 million) of all DMES spending. Of those patients with a chronic condition, a smaller percentage (14%) used ophthalmologic DMES.

Across the 786 specific chronic conditions experienced by pediatric patients in the cohort, the median (IQR) percentage of DMES use by individual chronic condition was 32.7% (IQR, 20.5%-50.0%). Examples of chronic conditions with DMES use in the 75th percentile and greater (DME use range for these chronic conditions: 50% to 100% of children with the conditions) included cerebral palsy, cystic fibrosis, quadriplegia, lysosomal storage diseases (eg, Tay-Sachs, mucolipidosis, sphingolipidosis), neuromuscular scoliosis, spina bifida, spinal muscular atrophy, and trisomy 13 and 18. Examples of chronic conditions in the 25th percentile and lower (DME use range: 0.0% to 20.5% for children with the conditions) included sickle cell anemia, juvenile idiopathic arthritis, neurofibromatosis, and von Willebrand disease. Other examples of DMES use by chronic condition included 41.3% for hypogammaglobinemia 41.6% for trisomy 21; 34.5% for epilepsy, and 20.9% for regional enteritis and ulcerative colitis.

### DMES Use and Spending by Chronic Condition Count and Complexity

Use rates of DMES were significantly higher in pediatric patients with a greater number of chronic conditions and higher complexity, ranging from 20.8% for patients with 1 noncomplex chronic condition to 60.1% for patients with 2 or more complex chronic conditions (CCCs)(*P* < .001) (Figure 2). The DMES use was similar in patients with 2 noncomplex chronic conditions and patients with 1 CCC (32.5% vs 33.5%; *P* = .10). The PMPY DMES spending ranged from a $378 for patients with 1 noncomplex chronic condition to $4253 for children with 2 or more CCCs (Figure 2).

Use of multiple DMES was most common in pediatric patients with 2 or more CCCs. For example, the prevalence of DMES use across 3 or more organ system categories increased from 0.9% in patients with one noncomplex chronic condition to 32.9% in children with 2 or more CCCs, *P* < .001. The most common DMES organ system categories used by patients with 2 or more CCCs were musculoskeletal (24.7%), nutrition (24.4%), genitourinary (21.4%), and respiratory (18.0%) (Table 2). Among the most common DMES types in these patients were enteral supplies (19.8%), diapers (19.2%), formula (18.7%), enteral tube (13.3%), and lower extremity orthotic (12.3%). In these patients, DMES types with the highest PMPY spending were parenteral supplies (PMPY $92 531), parenteral nutrition (PMPY $52 680), tracheostomy (PMPY $20 795), infusion pump (PMPY $14 724), and wound drainage (PMPY $14 585) (Table 2).

**Table 2.** Prevalence and Spending of DMES in Pediatric Patients With Multiple Complex Chronic Conditions Who Use Medicaid

DMES variable	No. (%) or PMPY spending, US $
**DMES organ system category, No. (%)**	
Musculoskeletal	12 919 (24.7)
Nutrition	12 762 (24.4)
Genitourinary	11 193 (21.4)
Respiratory	9415 (18.0)
Other	94049 (17.3)
Ophthalmologic	8526 (16.3)
Gastroenterological	8107 (15.5)
Skin and soft tissue	5649 (10.8)
Vascular	1778 (3.4)
Ear, nose, and throat	1465 (2.8)
Endocrine	523 (1.0)
Neurologic	105 (0.2)
Renal	105 (0.2)
Cardiac	52 (0.1)
**Most common DMES types, No. (%)**	
Enteral supplies	10 359 (19.8)
Diapers	10 043 (19.2)
Formula	9781 (18.7)
Frame lens	8369 (16.0)
Vision frames	8107 (15.5)
Enteral tube	6957 (13.3)
Lower extremity orthotic	6434 (12.3)
Underpads	5754 (11.0)
Skin dressing	5231 (10.0)
Wheelchair	5021 (9.6)
Nebulizer	4655 (8.9)
Oxygen	4132 (7.9)
Suction	3661 (7.0)
Gloves	3609 (6.9)
**DMES types with highest spending, US $**	
Parenteral supplies	92 531
Parenteral nutrition	52 680
Tracheostomy	20 795
Infusion pump	14 724
Wound (drainage)	14 585
Enteral supplies	12 163
Oxygen	11 824
Ostomy	10 703
Suction	9829
Glucometer	9012
Insulin pump	8869
Urinary catheterization supplies	8738
Infusion pump (NOC)	8460
Ventilator	8133

Abbreviations: DMES, durable medical equipment and supplies; NOC, not otherwise classified; PMPY, per-member-per-year spending.

## Discussion

In this cross-sectional study, the categorization of HCPCS codes identified a variety of DMES types associated with a wide range of Medicaid spending. The categorized system sufficiently differentiated the DMES types and related spending across different pediatric populations, including those with no chronic conditions and those with multiple CCCs. Vision lenses/frames and orthopedic splints and braces were among the most common DMES used in otherwise healthy patients, whereas enteral tube supplies, nutritional formula, and oxygen were among the most common DMES used in patients with multiple CCCs. The categorized HCPCS system reported the highest rates of DMES use in pediatric chronic conditions with expected high use, including quadriplegia and spinal muscular atrophy. Overall, the initial development and face validity testing of the categorized HCPCS codes in the current study shows potential to advance knowledge about DMES use in pediatric patients.

Prior literature^14^ substantiates the DMES prevalences reported in the current study. For example, the percentage of pediatric patients with claims for the most common DMES—eyeglasses and corrective lenses - in the current study (11%) is similar to the same percentages of use reported in the National Survey of Children’s Health (3% [for ages 2-to-5 years] to more than 35% [for ages 12-17 years]). In the measurement of DMES prevalence through HCPCS health care claims, it is important to recognize that prescribed DMES cannot be detected if they were dispensed before the study period of measurement or if they have not been yet dispensed. Regarding ophthalmologic DMES, socioeconomically disadvantaged children using Medicaid are the most likely to have unmet eye care needs; up to 1 in 7 parents are unable to obtain prescribed eyeglasses or corrective lenses for their children, due to a variety of challenges, including accessibility to eye care.^15,16^ Therefore, HCPCS health care claims may undercount the true population need for a particular DMES as well as the population prevalence of the underlying health problem that necessitates DMES use.

The current study highlights the need for medical formula as one of the most common DMES used in infants and children enrolled in Medicaid. Medicaid covers infant formula for low-income families in coordination with USDA’s Food and Nutrition Service’s Special Supplemental Nutrition Program for Women, Infants and Children.^17^ Medicaid also provides direct payment for formula prescribed by clinicians for CYSHCN, including those with special formula needs due to an underlying chronic illness (eg, metabolic disease) and those requiring formula for gastrostomy and related routes of nutrition. Given the 2022 recall of certain infant formulas, supply chain issues, and closure of a prominent formula-producing plant contributed to the recent US national shortage of nutritional formulas, precise categorization of patients at high risk for supply failures is critical and this DMES categorization may be useful to identify at-risk patients should other supply failures occur in the future.^18^

To our knowledge, the current study is the first to report that enteral tube and feeding supplies were the most common DMES dispensed in the home for pediatric patients with multiple CCCs. Used by 1 in 5 patients with multiple CCCs, these DMES include gastrostomy and jejunostomy tubes, syringes, set tubing, and infusion pumps as well as enteral feeding tube kits. The high prevalence of home enteral tube and feeding DMES is consistent with prior studies reporting a high rate of gastrostomy among hospitalized children with multiple CCCs who are enrolled in structured clinical programs for children with medical complexity.^19^ The high prevalence from the current study is also supported by our clinical experience that patients with multiple CCCs are at risk for oropharyngeal dysphagia, gastroesophageal reflux, aspiration pneumonia, sialorrhea, malnutrition, and other health problems that necessitate bypassing the oropharynx for the route of nutrition intake.

### Limitations

This study has several limitations. The sample of pediatric patients continuously enrolled in Medicaid had a high prevalence of chronic conditions, which is a factor in DMES use. The use of DMES may be lower in noncontinuously enrolled patients; this warrants investigation. The prevalence of DMES was likely undercounted, as DMES claims that occurred before the study period were not assessed (eg, power wheelchair DMES claimed in prior years without subsequent claims for adjustments or components). To our knowledge, the database does not include enrollees using both commercial insurance and Medicaid. The DMES obtained not through Medicaid (eg, loan from an early intervention agency, school, or other state-based program) were not measurable. DMES dispensed as part of an inpatient stay may not have been distinguished in the health care claim (eg, vagal nerve stimulator placed in an operating room). Therefore, the DMES findings in the current study cannot capture all patients assisted with medical technology. The current study was not positioned to measure daily concurrent use of DMES, duration of DMES use (eg, short vs long-term) or amount of DMES use (eg, NIPPV while sleeping vs continuous).

There is variation in the quality of Medicaid data across states, especially data for enrollees in managed care plans.^20^ The data usage agreement for the Medicaid claims data blinded the US states included in the study. Therefore, we were unable to assess the representativeness or generalizability of the sample for Medicaid as a whole. Some DMES claims might have been bundled (ie, multiple DMES submitted as one claim with one aggregate payout from Medicaid), which could affect estimates of spending for individual DMES. Although unavailable, Medicaid payment received by DMES vendors may be more accurate, especially for DMES spending that was estimated for managed care enrollees. The Agency for Healthcare Research and Quality Chronic Condition Indicator System does not include amblyopia, strabismus, myopia, and other related ophthalmologic diagnoses as a chronic condition; this may help explain use of DMES in patients without a coded chronic condition.

## Conclusions

Results of this cross-sectional study suggest that use of HCPCS to report DMES prevalence, types, and spending in pediatric patients with Medicaid in the current study is an important first step to advance knowledge on DMES use in pediatric patients. A variety of subsequent reliability and validity testing of HCPCS, including correlation of DMES claims with actual use) will be necessary to refine and optimize the system. However, our ultimate hope is that the HCPCS categorization and corresponding study findings will prompt subsequent investigations that will optimize the quality of care and clinical outcomes of patients using DMES. Examples of such investigations include, but are not limited to, (1) assessment of DMES prescription-to-delivery timing and efficiency; (2) measurement and root-cause analysis of DMES errors and adverse events across different types of DMES; (3) state-level health system evaluations of supply and demand related to the population size of patients using DMES, case management and home health care workforce, and number of DMES vendors; (4) applicability of DMES to refine risk-adjustment algorithms for suboptimal clinical outcomes (eg, acute health demise) as well as increased health care utilization and spending; (5) comparative effectiveness studies of DMES use with improvements and gains in functional status (eg, preoperative use of NIPPV and in-exsufflator in patients with neuromuscular conditions); (6) use of DMES information to distinguish and advocate for resources to support patients with the greatest need for enhanced care coordination and complex care services; and (7) contribution of DMES types (including use of multiple DMES) on family caregiving burden. We hope that the DMES classification schema tested in the current study will help enable these investigations and advance knowledge on DMES use in pediatric patients.

## Notes

Supplement 1. **eTable 1.** HCPCS DME Summary **eTable 2.** HCPCS DME Full Categorization

Supplement 2.
eAppendix.

Supplement 3.
Data Sharing Statement
