# Supplementary material for: Categorization of a Universal Coding System to Distinguish Use of Durable Medical Equipment and Supplies in Pediatric Patients
Source: JAMA Netw Open. 2023 Oct 24;6(10):e2339449. doi: 10.1001/jamanetworkopen.2023.39449 (PMC10599121; doi:10.1001/jamanetworkopen.2023.39449)
Supplement: Supplement 2. — eAppendix. [file jamanetwopen-e2339449-s002.pdf]

# Supplemental Online Content

Hotz A, Sprecher E, Bastianelli L, et al. Categorization of a universal coding system to distinguish use of durable medical equipment and supplies in pediatric patients. *JAMA Netw Open*. 2023;6(10):e2339449. doi:10.1001/jamanetworkopen.2023.39449

**eAppendix.** Technical Details on DMES Categorization Methods

This supplemental material has been provided by the authors to give readers additional information about their work.

© 2023 Hotz A et al. *JAMA Network Open*.

**eAppendix.** Technical Details on DMES Categorization Methods

## Durable Medical Equipment and Supply Categorization of the Healthcare Common Procedure Coding System

### Background of the Healthcare Common Procedure Coding System

The Healthcare Common Procedure Coding System (HCPCS) is a collection of health administrative billing codes that is stewarded by the Centers for Medicare and Medicaid Services (CMS). The codes are used for billing and reimbursement of health services and treatments rendered to patients. HCPCS is the coding system used by durable medical equipment and supply (DMES) companies for receipt of DMES including those delivered for use in patients' homes. HCPCS also includes codes for procedures and specialized health services, including home health care and therapies. For further information about HCPCS codes, go to https://www.cms.gov/Medicare/Coding/MedHCPCSGenInfo.

HCPCS is organized into 17 sections, denoted with the letters for related services and treatments. Within each HCPCS letter categorization, similar types of specific services and treatments are listed in proximity. Although many DMES HCPCS codes reside in HCPCS Section E, most codes for DMES are spread throughout a variety of other HCPCS sections. For example, of the 11 HCPCS codes for home mechanical ventilator, 7 are in Section E and 4 are in other sections. HCPCS does not categorize specific DMES types (e.g., it does not categorize these 11 codes as their common purpose as "ventilator") and it does not assign the codes to a body system (e.g., ventilator is not categorized as part of a larger category, such as 'respiratory body system').

The purpose of our HCPCS categorization is to distinguish specific types of DMES and assign each specific type to a body system. Creating a detailed DMES classification system will permit clinicians, researchers, administrators, payors, and others to better understand the populations of patients who use DMES, while focusing on the subsections that are most relevant to their practice or interests.

Visit the Children's Hospital Association website, Toolkit section, for access to updated files for the HCPCS categorization: https://www.childrenshospitals.org/content/analytics/toolkit/durable-medical-equipment-and-supply-categorization-of-the-healthcare-common-procedure-coding-system.

### Definition of Durable Medical Equipment and Supplies

HCPCS items that were considered DMES were included for categorization if they represented equipment or supplies intended to address a medical, nutritional, sensory, and/or functional need. Items could be dispensed from any health service (e.g., DMES vendor, pharmacy, hospital) and prescribed by any health care professional. Item use could be acute or chronic as well as continuous or episodic. Item use could be applicable to all pediatric populations, including healthy children and children with medical complexity. Although the categorization occurred with a focus on children, no DMES were excluded if used predominately by adult patients.

Medications and health services were not considered DMES and were excluded from the categorization in some circumstances:

- In general, medications – including infusions - were not considered DMES. For instance, immune globulin (HCPCS J1575) was not included. However, the DMES needed for

© 2023 Hotz A et al. *JAMA Network Open*.

immune globulin infusion at home (e.g., port, needle, tubing) was included. Oxygen was a notable exception. Oxygen was included as DMES and not excluded with other medications because it is typically considered as DMES and it is integrally related to other DMES for administration, including concentrator, tank, tubing, nasal cannula, mask, oximeter, etc.

- HCPCS health service codes were included as DMES only if they included specific DMES items [e.g., HPCPS S9343: Home therapy; enteral nutrition via bolus; administrative services, professional pharmacy services, care coordination, and all necessary supplies and equipment (enteral formula and nursing visits coded separately), per diem]. HCPCS health service codes were included as DMES if they described modification or repairs of existing DMES items in use (e.g., HCPCS V5336: Repair/modification of augmentative communicative system or device) as the code may indicate active use of an item.

- In this version of HCPCS categorization, dental-related DMES, such as retainers, were not included. We did this to focus primarily on other medical-related DMES, although inclusion of these codes could be considered in future iterations of the categorization system.

- For the current HCPCS categorization, most HCPCS codes referring to equipment used during a surgery or related procedure (e.g., equipment and supplies used for cardiac catheterization) were not included as DMES. Exceptions occurred with implanted devices intended for extended, non-transient use that addressed a deficit in physiologic function [e.g., HCPCS C1721: cardioverter-defibrillator, dual chamber (implantable)].

Special considerations were also made for vision and nutritional formula DMES:

- All HCPCS codes for sensory DMES were included for categorization, including DMES for vision and hearing. Thus, DMES for corrective lenses, eyeglasses, and frames were included. These DMES were categorized in the Ophthalmologic category, enabling feasibility to distinguish the HCPCS codes and include or exclude them depending on the purpose and scope of a particular project.

- HCPCS codes for nutritional formulas were also included as DMES, including specialized formulas (e.g., for inborn error of metabolism). Formula was included given that insurers and state Medicaid programs often consider formula as DMES and will pay for specialty formulas if medically necessary. Formula DMES were categorized in the nutrition category, enabling feasibility to distinguish the HCPCS codes and include or exclude them depending on the purpose and scope of a particular project.

© 2023 Hotz A et al. *JAMA Network Open*.

With these considerations, 2576 HCPCS codes that represent DMES were identified, based on the definitions and exclusions/inclusions described above. The number of HCPCS codes distinguished as DMES by HCPCS Category Letter is presented in the table below.

**Number of HCPCS Codes Identified as DMES by HCPCS Category**

| HCPCS Category Letter | HCPCS Category Description | No. of HCPCS Codes Identified as DMES |
|---|---|---|
| A | Transportation services including ambulance, medical and surgical supplies | 468 |
| B | Enteral and parenteral therapy | 48 |
| C | Temporary codes for use with outpatient prospective payment programs | 42 |
| D | Dental | 0 |
| E | Durable medical equipment (DME) | 578 |
| G | Procedures/professional services (Temporary Codes) | 0 |
| H | Alcohol and Drug Abuse Treatment Services / Rehabilitative Services | 0 |
| J | Drugs administered other than oral method, chemotherapy drugs | 0 |
| K | Durable medical equipment for Medicare administrative contractors | 136 |
| L | Orthotic and prosthetic procedures, devices | 888 |
| M | Medical services | 0 |
| P | Pathology and laboratory services | 0 |
| Q | Miscellaneous services (Temporary codes) | 85 |
| R | Diagnostic radiology services | 0 |
| S | Commercial payers (Temporary codes) | 121 |
| T | Established for state medical agencies | 28 |
| V | Vision, hearing, and speech language services | 182 |

© 2023 Hotz A et al. *JAMA Network Open*.

## Categorization of HCPCS Codes by DMES Types and Systems

Each DMES code was assessed for categorization to an end-organ category (e.g., cardiac, neurologic, renal). Because nutritional formula was given special consideration as DMES, formula HCPCS codes were assigned to the nutrition system. The DMES codes not assignable to an end-organ or system category were categorized as "other." Examples of "other" DME with these attributes include batteries, gloves, needles/syringes, and sterile water. Many of these categories could apply or support DMES found in other categories.

Two complex care pediatricians initially categorized the DMES codes distinguished in HCPCS by DMES type and end-organ system. A third pediatrician adjudicated discrepancies in category assignments. The pediatricians then created a multi-disciplinary, DMES stakeholder panel, with panelist expertise sought from the clinical content of the initial end-organ system categories, as well as the respective knowledge of specific DMES types associated with each end-organ system. Fifteen panelists were recruited and all participated.

Panelists included medical and surgical generalists as well as a physical therapist, a dietician, a family partner, case manager / insurance specialist. Panelists were instructed to initially focus on the DMES types and system categorization of the end-organ that matched best to their expertise (e.g., the pediatric pulmonology specialist reviewed respiratory-related DMES and categories). Then, panelists were instructed to review the full HCPCS system, and were encouraged to review and suggest revisions for DMES codes and categories beyond their specialty. Specific tasks charged to the panelists included (1) identification of DMES types missing from the category of their expertise; (2) evaluation of each DMES type in their category to ensure that the type was not better categorized elsewhere; (3) evaluation of the name (i.e., label) for each DMES type and category.

Panelist revisions included: (a) 151 of 2576 total DMES codes (6%) changed to a different end-organ system category, suggesting 94% agreement with the initial DME system categorization; (b) 205 DMES codes (8%) changed to a different or new DMES type within an end-organ system category, suggesting 92% agreement with DMES type categorization; (c) wording revision to the name of the category associated with 218 DMES codes (8%).

© 2023 Hotz A et al. *JAMA Network Open*.

**DMES Systems and Number of Corresponding HCPCS Codes**

| System Category | No. of HCPCS Codes | | System Category | No. of HCPCS Codes |
|---|---|---|---|---|
| Cardiac | 56 | | Nutrition | 56 |
| Ear, Nose, and Throat | 120 | | Ophthalmologic | 149 |
| Endocrine | 33 | | Renal | 7 |
| Gastroenterologic | 81 | | Respiratory | 164 |
| Genitourinary | 93 | | Skin and soft tissue | 149 |
| Musculoskeletal | 1409 | | Vascular | 98 |
| Neurologic | 37 | | Other | 124 |

***Specific DMES Type and System Categorization***

**<u>Cardiac</u>**
**Total # of codes:** 56

**This system includes**: DMES that support the heart, such as pacemakers, ventricular assist device.

**Considerations with classification:**

- Compression stockings, infusion supplies are located in the "Vascular" System.
- Blood pressure cuffs are classified in the "Renal" System.

**Cardiac**

| DME Type | No. of codes |
|---|---|
| Defibrillator (External) | 5 |
| Defibrillator (Internal) | 6 |
| Implantable Rhythm Recorder | 2 |
| Pacemaker | 10 |
| Pacemaker/Defibrillator | 1 |
| Ventricular Assist | 32 |

**<u>Ear, Nose and Throat</u>**
**Total # of codes**: 120

**This system includes:** hearing aides, cochlear implants and augmented communication devices.

**Considerations with classification:**
- DMES supporting tracheostomy and supplies were classified with the Respiratory system, to keep those DMES with others that support breathing, oxygenation, and ventilation.
- DMES that support augmented communication, support expressive speech and communication, including trach-related speech, are included in this section.

**Ear, Nose, and Throat**

| DME Type | No. of codes |
|---|---|
| Artificial Larynx | 2 |
| Assisted Hearing | 64 |
| Bone Anchored Hearing Aides | 5 |
| Brainstem Implant | 1 |
| Cochlear Implant | 14 |
| Joint Device (Face) | 3 |
| Oral Appliance | 2 |
| Prosthesis (Face) | 8 |
| Pulse Generator System | 2 |
| Speech Generating Device | 11 |
| Tracheoesophageal Voice Prosthesis | 7 |
| Voice Amplifier | 1 |

© 2023 Hotz A et al. *JAMA Network Open*.

**Endocrine**
**Total # of codes:** 33

**This system includes:** DMES primarily involving diabetes care.

**Considerations with classification:**
- Injectable supplies, such as those used for growth hormone, are assigned to the "Other" system as injectable supplies are used for non-endocrine medications as well.

**Endocrine**

| DME Type | No. of codes |
|---|---|
| Artificial Pancreas | 4 |
| Continuous Glucose Monitoring | 7 |
| Glucometer | 14 |
| Insulin Pump | 7 |
| Ketone Monitoring | 1 |

**Gastroenterologic**
**Total # of codes:** 91

**This system includes:** DMES that support enteral feeding and ostomies.

**Considerations with classification**:
- Ostomy codes were assigned to the Gastroenterologic system unless specifically designated for urinary ostomies.
- Formulas, fluids, and parenteral nutrition are assigned to "Nutrition."
- HCPCS code S8265, "Haberman feeder for cleft lip/palate," was categorized Gastrointestinal section as opposed to Ear Nose Throat as it addresses feeding.

**Gastroenterologic**

| DME Type | No. of codes |
|---|---|
| Bowel Irrigation | 5 |
| Enteral Tube | 2 |
| Enteral Supplies | 10 |
| Gastric suction | 2 |
| Gastroenterologic Ostomy | 67 |
| Haberman Feeder | 1 |
| Nasogastric Tube | 2 |
| Perianal stool collection | 1 |
| Scale | 1 |

**Genitourinary**
**Total # of codes:** 93

**This system includes:** DMES that support incontinence and catheterization.

**Considerations with classification:**
- Some of these codes, for instance Prosthesis (Genitourinary), erectile device and pessary, are unlikely to be used often among pediatric codes. However, these were included in effort to be as inclusive as possible.
- Incontinence (Not otherwise classified) includes nonspecific codes such as named A4335 for "incontinence supply."

**Genitourinary**

| DME Type | No. of codes |
|---|---|
| Bedpan | 2 |
| Diapers | 20 |
| Enuresis Alarm | 1 |
| Erectile Device | 2 |
| Incontinence (Not Otherwise Classified) | 3 |
| Pessary | 2 |
| Prosthesis (Genitourinary) | 3 |
| Underpads | 7 |
| Urinary Cathing | 29 |
| Urinary Ostomy | 19 |
| Urine Collection | 5 |

© 2023 Hotz A et al. *JAMA Network Open*.

**<u>Musculoskeletal</u>**
**Total # of codes:** 1409

**This system includes:** DMES that support function, such as equipment to support ambulation, orthotics, prostheses, and wheelchairs.

**Considerations with classification:**

- Beds and mattresses were included in this section as a physical therapist is often involved in these prescriptions.
- "Musculoskeletal Not Otherwise Classified" includes codes that don't fit elsewhere, such as A9281 "reaching/grabbing device", and E0945 "belt/harness extremity."
- "Ambulation Not Otherwise Classified" includes a code that is not specific to the other ambulation devices, A4637 "Replacement, tip, cane, crutch, walker, each."
- "Orthotic Not Otherwise Classified" includes codes that could be general to any orthotic, such as L4002 "replacement strap, any orthosis." Also included is a L4210, a code for "Repair of orthotic device, repair or replace minor parts." While repairs are generally considered a service and are not included, this type of bundled code that includes the parts themselves are included.
- "Prosthesis Not Otherwise Classified" includes parts that could be used for multiple different prostheses, such as L7600, which is a "prosthetic donning sleeve" or L7700 for a "gasket or seal for use with prosthetic insert."

**Musculoskeletal**

| DME Type | No. of codes |
|---|---|
| Ambulation (Cane) | 3 |
| Ambulation (Forearm Crutch) | 3 |
| Ambulation (Not Otherwise Classified) | 1 |
| Ambulation (Walker) | 15 |
| Bathing / Toileting | 21 |
| Gait Trainer | 3 |
| Helmet | 6 |
| Joint Device (Lower Extremity) | 9 |
| Joint Device (Upper Extremity) | 9 |
| Mattress Bed | 50 |
| Musculoskeletal (NOC) | 4 |
| Orthopedic Implant | 6 |
| Orthopedic Injury | 80 |
| Orthotic (Cervical) | 14 |
| Orthotic (Foot) | 98 |
| Orthotic (Lower extremity) | 174 |
| Orthotic (NOC) | 2 |
| Orthotic (Spinal) | 96 |
| Orthotic (Upper Extremity) | 65 |
| Osteogenesis Stimulator | 4 |
| Powered Operated Vehicle | 1 |
| Prosthesis (Lower Extremity) | 197 |
| Prosthesis (NOC) | 5 |
| Prosthesis (Upper extremity) | 150 |
| Seating | 1 |
| Standing | 3 |
| Traction | 17 |
| Traction (Halo) | 5 |
| Transfer System | 18 |
| Transport Chair | 4 |
| Wheelchair | 345 |

© 2023 Hotz A et al. *JAMA Network Open*.

**<u>Neurologic</u>**
**Total # of codes:** 37

**This system includes:** DMES for neurostimulators and related leads.

**Considerations with classification**:
- “Neurostimulator (NOC)” included codes that did not specify if a neurostimulator was internal or external.

**Neurologic**

| DME Type | No. of codes |
|---|---:|
| Electromyography | 1 |
| Intraspinous Infusion Pump | 1 |
| Neurostimulator (External) | 17 |
| Neurostimulator (Implantable) | 16 |
| Neurostimulator (Not Otherwise Classified) | 2 |

**<u>Nutrition</u>**
**Total # of codes:** 34

**This system includes:** enteral formula, fluids and supplements as well as parenteral nutrition.

**Considerations with classification:**
- DMES related to infusions that were not specified for nutrition were assigned to the Vascular system.
- Formula itself is in this section, but the supplies related to formula (e.g., gastrostomy tube, enteral supplies) were assigned to the Gastroenterology system.
- Parenteral Nutrition codes are located in this section, but the supplies related to administration of parenteral nutrition are located in the vascular section.
- Medical food refers to solid food supplements designed and dispensed for patients with special diets related to inborn errors of metabolism.

**Nutrition**

| DME Type | No. of codes |
|---|---:|
| Enteral Rehydration Fluid | 2 |
| Formula | 8 |
| Formula (Metabolic) | 4 |
| Medical Food | 2 |
| Nutrition (Additive) | 2 |
| Nutrition (Thickener) | 1 |
| Parenteral Nutrition | 15 |

**<u>Ophthalmologic</u>**
**Total # of codes:** 148

**This system includes:** lens, frames, and eye prostheses.

**Considerations with classification:**
- “Prosthesis (Face)” in the ENT section and the Ophthalmologic section does not include orbital prosthesis.
- “Eye NOC DMES” includes V2756 “Eye glass case” and V2799 “Vision item or service, miscellaneous.”

**Ophthalmologic**

| DME Type | No. of codes |
|---|---:|
| Contact Lens | 16 |
| Eye (Not Otherwise Classified) | 3 |
| Eye Implant | 19 |
| Eye Pad | 2 |
| Eye Patch | 1 |
| Frame Lens | 89 |
| Low Vision Aides | 3 |
| Ocular Adenexal Implant | 1 |
| Prosthesis (Eye) | 7 |
| Prosthesis (Orbital) | 1 |
| Scleral Lens | 3 |
| Vision Frames | 4 |

© 2023 Hotz A et al. *JAMA Network Open*.

**Other**
**Total # of codes:** 124

**This system includes:** DMES which did not have a more appropriate assignment to one of the remaining systems.

**Considerations with classification:**

- Many of the “Other” system DMES have multiple uses across multiple systems (e.g., needles, catheter NOC) and/or do not have sufficient specificity for routing to another system. An example of this is code L7367 “Lithium ion battery, rechargeable, replacement).”
- “Other (Not otherwise classified)” includes very general codes, such as T5999 “Supply, not otherwise specified” and E0700 “Safety equipment, device or accessory, any type.”
- “Prosthesis not otherwise” includes nonspecific codes such as L8699 “Prosthetic implant, not otherwise specified,” as this does not clearly indicate a specific area of the body it is supporting.

**Other**

| DME Type | No. of codes |
|---|---|
| Alarm (Not Otherwise Classified) | 1 |
| Batteries (Not Otherwise Classified) | 7 |
| Belt | 2 |
| Binders | 1 |
| Breast Pump | 9 |
| Electromagnetic Device | 1 |
| Gel Paste | 2 |
| Gloves | 1 |
| Infusion Pump (External) | 10 |
| Infusion Pump (Implantable) | 8 |
| Infusion Pump (Not Otherwise Classified) | 5 |
| Injectable Supplies | 6 |
| Irrigation | 2 |
| Lubricant | 2 |
| Needles / Syringes | 11 |
| Not specified | 6 |
| Other (Not Otherwise Classified) | 7 |
| Paraffin | 1 |
| Post Mastectomy | 15 |
| Prosthesis (Not Otherwise Classified) | 1 |
| Restraints | 1 |
| Sterile Water | 2 |
| Thermometer | 1 |
| Truss | 4 |
| Warming/Cooling Device | 15 |
| Whirlpool | 2 |
| Wig | 1 |

© 2023 Hotz A et al. *JAMA Network Open*.

**Respiratory**
**Total # of codes:** 164

**This system includes:**
aerochamber, nebulizer, trach-related supplies, ventilators.

**Considerations with classification:**

- "Tracheostomy" is classified as Respiratory, not under "Ear, Nose and Throat," so that it resides with other DMES that support breathing, oxygenation, and ventilation.
- "Ventilation (NOC)" refers to general codes that could support either ventilator or non-invasive ventilation.
- "Respiratory Not Otherwise Classified" includes nonspecific respiratory codes that could fit under multiple other categories, such as a "mouth piece" or a "swivel adaptor."

**Respiratory**

| DME Type | No. of codes |
|---|---|
| Aerochamber / Peak Flow | 6 |
| Airway Clearance | 13 |
| Apnea Monitor | 4 |
| Chest Tube | 4 |
| Nebulizer | 25 |
| Non-invasive Negative Pressure Ventilation | 3 |
| Non Invasive Positive Pressure Ventilation | 15 |
| Oximeter | 2 |
| Oxygen | 33 |
| Respiratory (Not Otherwise Classified) | 2 |
| Resuscitation Bag | 1 |
| Spirometer | 2 |
| Suction | 6 |
| Tracheostomy | 29 |
| Vaporizer | 1 |
| Ventilator | 6 |
| Ventilation (Not Otherwise Classified) | 12 |

**Renal**
**Total # of codes:** 7

**This system includes:** dialysis-related codes and blood pressure cuffs.

**Renal**

| DME Type | No. of codes |
|---|---|
| Blood pressure | 2 |
| Dialysis | 5 |

**Considerations with classification:**

- 3 of these are c-codes related to implantable dialysis catheters and systems, and 2 are bundled codes related to home hemodialysis or peritoneal dialysis.
- Injectable supplies, such as those used for erythropoetin injections are categorized as vascular. As mentioned above, the medications themselves are not considered DMES.

**Skin and Soft Tissue**
**Total # of codes:** 149

**This system includes:** skin dressings, wound treatments.

**Considerations with classification:**

- The largest subcategory of this system includes DMES for "Skin Dressing," this may include gauze, hydrogel dressings, wound fillers, and compression bandages. These DMES were not further categorized because most of the DMES have multiple indications and uses for skin.

**Skin and Soft Tissue**

| DME Type | No. of codes |
|---|---|
| Burn Garment | 13 |
| Disinfectant | 4 |
| Hyperbaric Chamber | 2 |
| Phototherapy | 8 |
| Pressure Injury Prevention | 7 |
| Skin Dressing | 104 |
| Topical Oxygen | 1 |
| Wound (Drainage) | 9 |
| Wound (Other) | 1 |

© 2023 Hotz A et al. *JAMA Network Open*.

- “Phototherapy” was included in this system because it can be used to treat hyperbilirubinemia as well as to assist with wound healing.
- “Home hyperbaric oxygen” was included here because it is typically approved by insurance for an indication of wound healing.

**<u>Vascular</u>**
**Total # of codes:** 110

**This system includes:** Compression stockings, gradient pressure devices, infusion supplies (general and related to parenteral nutrition).

**Considerations with classification**:
- Supplies related to parenteral nutrition, but not the nutrition itself are included in this section.

**Vascular**

| DME Type | No. of codes |
|---|---|
| Compression Stocking | 20 |
| Gradient Pressure | 10 |
| Infusion Supplies | 48 |
| Indwelling Port | 2 |
| Parenteral Supplies | 12 |
| Pneumatic Compressor | 18 |

- While parenteral nutrition itself is categorized in the “Nutrition” Section, there are 4 codes included in the 12 “Parenteral supplies” subsection of “Vascular” that are bundled with the description including both the services and the supplies for infusion. In this case, as described above, bundled codes were included in an effort to not overlook when a DMES was being used.

© 2023 Hotz A et al. *JAMA Network Open*.

**Supplemental Table. Demographic and Clinical Characteristics of Pediatric Patients Enrolled in Medicaid With vs. Without Use of Durable Medical Equipment and Supplies.**

| Characteristic | | Durable Medical Equipment and Supply Use | |
|---|---|---|---|
| | No Durable Medical Equipment and Supplies Use N = 3,787,086 | Ophthalmologic Lens/Frames Only N = 468,494 | Other N =313,931 |
| **Age in Years** | | | |
| 0 | 33843 (0.9) | 26 (0.0) | 5174 (1.6) |
| 1 to 4 | 864501 (22.8) | 11545 (2.5) | 78095 (24.9) |
| 5-9 | 998609 (26.4) | 110244 (23.5) | 72112 (23.0) |
| 10-15 | 1068872 (28.2) | 217205 (46.4) | 94155 (30.0) |
| 16-18 | 472382 (12.5) | 94647 (20.2) | 38203 (12.2) |
| 19-21 | 348841 (9.2) | 34827 (7.4) | 26192 (8.3) |
| **Race/Ethnicity** | | | |
| White, non-Hispanic | 1750215 (46.2) | 234363 (50.0) | 156012 (49.7) |
| Black, non-Hispanic | 1334780 (35.2) | 143987 (30.7) | 93602 (29.8) |
| Hispanic | 283150 (7.5) | 42890 (9.2) | 23885 (7.6) |
| Other | 154590 (4.1) | 20058 (4.3) | 11534 (3.7) |
| Missing | 264313 (7.0) | 27196 (5.8) | 28898 (9.2) |
| **Sex** | | | |
| Male | 1948212 (51.4) | 200614 (42.8) | 167694 (53.4) |
| Female | 1838836 (48.6) | 267880 (57.2) | 146237 (46.6) |
| **Chronic Condition** | | | |
| No chronic condition | 2366404 (62.5) | 198886 (42.5) | 67023 (21.3) |
| Only 1 non-complex | 881641 (23.3) | 143264 (30.6) | 88515 (28.2) |
| 2+ non-complex | 372830 (9.8) | 90500 (19.3) | 89012 (28.4) |
| Only 1 complex chronic | 145742 (3.8) | 31228 (6.7) | 42123 (13.4) |
| 2+ complex chronic | 20431 (0.5) | 4616 (1.0) | 27258 (8.7) |

© 2023 Hotz A et al. *JAMA Network Open*.

```
/*******************************************************************************
*****************
Macro Name: DMES_V1

Function:
This program will be used to apply the DMES System & DMES Types to HCPCS codes

Author: Matt Hall, PhD; Children's Hospital Association

Date: August 18, 2023

Load macro:

%INCLUDE "\path\DMES_GROUPER_v1.sas";

Call statement:

%DMES_V1(dt_in,dt_out,pr);

Parameter definitions:

   dt_in:  SAS input data set containing patient id and a single HCPCS code
   dt_out: SAS output data set containing all input data and new created DMES
SYSTEM & DMES TYPE
   pr: variable name for the HCPCS code
*******************************************************************************
****************/

*PATHNAME specifies the location of the Excel file
"Hotz_HCPCS_DMES_System_8.15.23.xlsx";

%LET PATHNAME=\path;  *<===USER MUST modify;

/*Import DMES Excel*/
PROC IMPORT FILE="&PATHNAME.\Hotz_HCPCS_DMES_System_8.15.23.xlsx" DBMS=xlsx
OUT=DMES REPLACE;
        SHEET="HCPCS DME Full Categorization";
RUN;
%MACRO DMES_V1(dt_in,dt_out,pr);
        PROC SQL;
                CREATE TABLE &dt_out. AS
                SELECT &dt_in..*, DMES.*
                FROM &dt_in. LEFT JOIN DMES(KEEP=HCPCS_Code DMES_System DMES_Type)
                ON &dt_in..&pr.=DMES.HCPCS_Code;
        QUIT;
%MEND;
```

© 2023 Hotz A et al. *JAMA Network Open*.
