# Supplementary material for: Categorization of a Universal Coding System to Distinguish Use of Durable Medical Equipment and Supplies in Pediatric Patients
Source: JAMA Netw Open. 2023 Oct 24;6(10):e2339449. doi: 10.1001/jamanetworkopen.2023.39449 (PMC10599121; doi:10.1001/jamanetworkopen.2023.39449)
Supplement: Supplement 3. — Data Sharing Statement [file jamanetwopen-e2339449-s003.pdf]

Data Sharing Statement

Hotz. Categorization of a Universal Coding System to Distinguish Use of Durable Medical Equipment and Supplies in Pediatric Patients. *JAMA Netw Open*. Published October 24, 2023. doi:10.1001/jamanetworkopen.2023.39449

Data
Data available: No
